# GC-MS Profiling and Antimicrobial Activity of Eight Essential Oils Against Opportunistic Pathogens with Biofilm-Forming Potential

**DOI:** 10.3390/ijms262210928

**Published:** 2025-11-11

**Authors:** Ruxandra Ștefănescu, Eszter Laczkó-Zöld, Cristina Ciurea, Amelia Tero-Vescan, Bianca Ősz, Szende Vancea, Dragoș Sita, Anca Mare

**Affiliations:** 1 Department of Pharmacognosy and Phytotherapy, Faculty of Pharmacy, George Emil Palade University of Medicine, Pharmacy, Science, and Technology of Târgu Mureș, 540139 Târgu Mureș, Romania; ruxandra.stefanescu@umfst.ro; 2 Microbiology Department, George Emil Palade University of Medicine, Pharmacy, Science, and Technology of Târgu Mureș, 540142 Târgu Mureș, Romania; cristina.ciurea@umfst.ro (C.C.); anca.mare@umfst.ro (A.M.); 3 Department of Medical Chemistry and Biochemistry, Faculty of Medicine in English, George Emil Palade University of Medicine, Pharmacy, Science, and Technology of Târgu Mureș, 540139 Târgu Mureș, Romania; amelia.tero-vescan@umfst.ro; 4 Department of Pharmacology and Clinical Pharmacy, Faculty of Pharmacy, George Emil Palade University of Medicine, Pharmacy, Science, and Technology of Târgu Mureș, 540139 Târgu Mureș, Romania; bianca.osz@umfst.ro; 5 Legal Medicine Service, Emergency County Hospital Miercurea Ciuc, 530173 Miercurea Ciuc, Romania; vancsa.szende@gmail.com; 6 Department of Odontology and Oral Pathology, Faculty of Dental Medicine, George Emil Palade University of Medicine, Pharmacy, Science, and Technology of Târgu Mureș, 540139 Târgu Mureș, Romania; dragos.sita@umfst.ro

**Keywords:** essential oils, GC analysis, antibacterial activity

## Abstract

Essential oils (EOs) are complex plant-derived products known for their broad-spectrum antibacterial activity. This study aims to evaluate the chemical composition of eight essential oils-EOs (Caryophylli aetheroleum, Menthae aetheroleum, Origani aetheroleum, Rosmarini aetheroleum, Salviae aetheroleum, Melaleucae aetheroleum, Limonis aetheroleum, and Curcumae aetheroleum) and to evaluate their antibacterial and antibiofilm activity against five opportunistic pathogens with biofilm-forming potential (methicillin-susceptible and methicillin-resistant *Staphylococcus aureus*, *Pseudomonas aeruginosa*, *Enterococcus faecalis*, *Escherichia coli*, and *Klebsiella pneumoniae*). GC-MS was used to determine the chemical composition of the EOs, and antibacterial activity was evaluated using broth microdilution to determine the minimum inhibitory concentration and minimum bactericidal concentration. Biofilm inhibition was assessed by a crystal violet assay. Oxygenated monoterpenes and phenolic compounds were dominant in Origani, Menthae, Rosmarinus, Melaleucae, and Caryophylli aetheroleum. Potent inhibitory effects against the tested bacterial strains were observed for clove, tea tree, oregano, and rosemary EOs. The antimicrobial efficacy of EOs is closely linked to their chemical composition. Tea tree and oregano EOs exhibited the broadest spectrum of antimicrobial activity, while peppermint and curcuma oils were the least potent. Cytotoxicity thresholds from the literature suggest that some effective EO concentrations exceed safe mucosal limits, particularly in continuous high-dose applications, but short-contact delivery systems or adjunctive use with different agents may mitigate safety concerns. These findings support further investigation into their therapeutic applications in oral health products.

## 1. Introduction

Essential oils (EOs) are natural-derived products with very potent pharmacological activities [1,2,3]. The complex mixture in an essential oil is up to almost 100 different volatile compounds formed mainly from terpenes and phenylpropane derivatives with molecular weights lower than 300 Daltons [4]. However, out of the numerous compounds found in an essential oil, only two or three are considered to be major, while other compounds are only found in small or even trace amounts [2,5]. Among their various pharmacological activities, the antibacterial properties of essential oils have received increasing attention. Several EOs have demonstrated potent activity against a broad spectrum of bacteria, including both Gram-positive and Gram-negative species [6,7]. Despite their potential, the clinical application of essential oils as antibacterial agents remains limited and underexplored, particularly in the context of infectious diseases associated with oral health.

In this context, essential oils could offer a promising natural alternative for controlling pathogenic bacteria implicated in oral infections, and prevent microbial resistance. Recent evidence suggests that EOs may modulate host responses and microbial behavior through epigenetic and metabolic pathways, although this remains to be confirmed in vivo. Volatile compounds inhibit biofilm formation through direct bactericidal effects but also by interfering with bacterial communication (quorum sensing), disrupting extracellular polymeric substances, and altering surface adhesion properties [8,9].

This study aimed to investigate the chemical composition of eight selected essential oils using gas chromatography–mass spectrometry (GC-MS) and to evaluate their antibacterial activity against a panel of pathogens, including methicillin-susceptible and methicillin-resistant *Staphylococcus aureus* (MSSA and MRSA), *Pseudomonas aeruginosa*, *Enterococcus faecalis*, *Escherichia coli*, and *Klebsiella pneumoniae*. The essential oils were selected based on the literature evidence supporting their antimicrobial and anti-inflammatory effects, especially in the context of oral health. The oils also represent diverse chemotypes and dominant compound classes, allowing for a comparative analysis of their chemical profiles and their contribution to antibacterial activity. Moreover, several of these oils have shown synergistic effects when combined with conventional antibiotics or other compounds, justifying their inclusion in a formulation-oriented screening approach. The eight essential oils evaluated in this study: Tea tree (*Melaleuca alternifolia*), Clove (*Syzygium aromaticum*), Oregano (*Origanum vulgare*), Rosemary (*Rosmarinus officinalis*), Lemon (*Citrus limon*), Peppermint (*Mentha piperita*), Sage (*Salvia officinalis*), and Turmeric (*Curcuma longa*), are widely used in traditional medicine and cosmetic formulations. Their major bioactive compounds include terpinen-4-ol in tea tree oil, eugenol in clove, carvacrol and thymol in oregano, 1,8-cineole and camphor in rosemary, limonene in lemon, menthol in peppermint, thujone in sage, and ar-turmerone in turmeric. These constituents have demonstrated a broad spectrum of pharmacological effects, including antimicrobial, anti-inflammatory, antioxidant, and wound-healing properties [6,10,11,12,13,14,15]. We hypothesize that the antibacterial and anti-biofilm activity of the essential oils is closely linked to their chemical composition and that selected oils may demonstrate promising activity against opportunistic bacteria associated with oral dysbiosis.

## 2. Results

### 2.1. GC-MS Analysis of EO

After the GC-MS analysis, 12 compounds have been identified in *Syzygium aromaticum* EO, 23 in *Citrus limon* EO, eight in *Origanum vulgare* EO, 20 in *Mentha piperita* EO, 18 in *Rosmarinus officinalis*, 24 in *Salvia officinalis*, 16 in *Melaleuca alternifolia* oil, and 22 in *Curcuma longa* EO, as it can be seen in Table 1. The total peak area does not reach 100% for all samples because the compounds that were below the identification threshold (match factor < 85%) or under 0.1% relative abundance were not included in the reported data.

**Figure 1:**
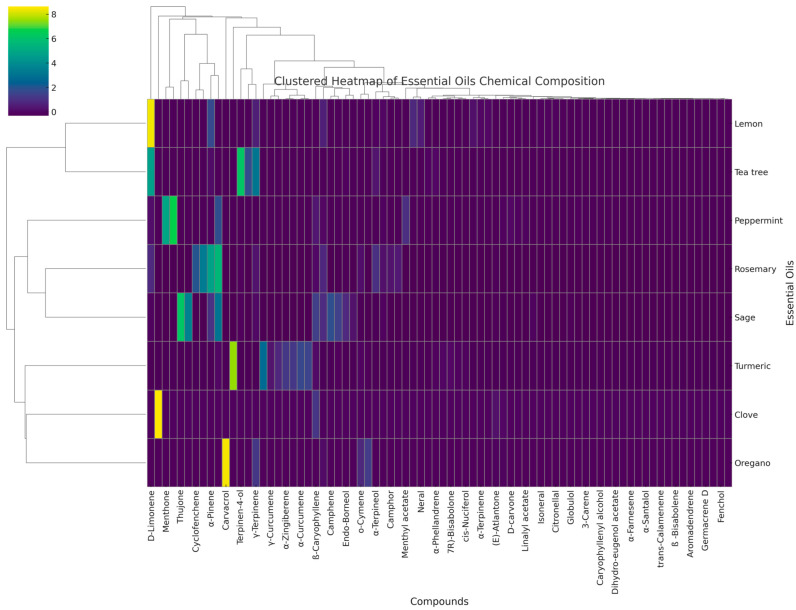
Hierarchical clustering of essential oils based on GC-MS profiles.

A hierarchical cluster analysis (HCA) was performed to evaluate the chemical similarity between the essential oils and their GC-MS profiles (Figure 1). Tea tree, peppermint, rosemary, and sage EOs displayed an intermediate clustering; this suggests that there is a partial overlap in their dominant compounds, while a separate cluster can be observed for lemon oil, which is dominated by monoterpene hydrocarbons (D-limonene), and for turmeric oil, which is dominated by oxygenated sesquiterpenes.

### 2.2. Antibacterial Effect

The MIC and MBC values determined in our study for MSSA (ATCC 29213), MRSA (ATCC 43300), *P. aeruginosa* (ATCC 27853), *E. coli* (ATCC 25922), and *K. pneumoniae* (ATCC 700603) are shown in Table 2.

On MSSA tea tree, rosemary, sage, and oregano EO were the most potent, while on MRSA tea tree, clove, rosemary, and oregano. *Pseudomonas aeruginosa* was the least susceptible to the EOs; however tea tree and clove were the most efficient. On *E. coli*, oregano, clove, rosemary, and tea tree showed MIC values lower that 3.125%, while on *Klebsiella pneumoniae*, only tea tree showed lower MIC values, but activity was also recorded for rosemary, lemon, clove, and oregano.

Overall, out of the tested essential oils, tea tree, clove, rosemary, and oregano exhibited significant potent activity regarding the MIC and MBC concentrations. Surprisingly, peppermint oil was the least potent EO on the tested bacterial strains.

Regarding the biofilm inhibition, notable differences were observed in the efficacy of the tested essential oils (EOs) against the selected bacterial strains (Table 3).

Among the Gram-positive isolates, the formation of biofilms by methicillin-susceptible *Staphylococcus aureus* (MSSA) was significantly inhibited by the essential oils of sage, oregano, lemon, and tea tree. In contrast, biofilm formation by methicillin-resistant *Staphylococcus aureus* (MRSA) proved to be more resistant, with only lemon and tea tree essential oils demonstrating inhibitory activity.

Because the MIC values for some essential oils were above 1%, a basic in silico toxicity screening was performed for the main compounds found in the eight essential oils. The predicted oral LD_50_ (Table 4) values were within acceptable ranges, supporting their potential use in formulations at sub-toxic concentrations.

## 3. Discussion

Oral infections are prevalent among the adult population, and although often considered minor, these are frequently underestimated or inadequately treated. Delayed intervention or neglect can allow the progression of the infection, leading to chronic inflammation, systemic complications, or the emergence of antimicrobial resistance. Biofilms serve as protective environments for bacteria, making them resistant to antimicrobial agents and host immune responses. The presence of multidrug-resistant bacteria within these biofilms complicates treatment efforts and potentially leads to chronic infections and disease recurrence [22]. Therefore, preventive strategies aimed at maintaining oral health are very important. Prophylactic measures, such as regular dental check-ups, effective oral hygiene practices, and early intervention in cases of oral infections, remain the most effective methods of reducing the risk of disease onset and progression. In these prophylactic measures, the use of natural-derived products could be an option, due to the low risk of side effects. Among the natural-derived products, essential oils have a great potential to be used as a prophylactic measure due to their multiple effects, with antibacterial action being the most known. The specificity of the antibacterial action of EOs is linked with their chemical composition, as previous studies have demonstrated that EOs that are rich in phenols and alcohols have the best antibacterial profile [9,12,23].

*Curcuma longa*, *Melaleuca alternifolia*, *Mentha piperita*, *Origanum vulgare*, *Rosmarinus officinalis*, *Syzygium aromaticum*, and *Salvia officinalis* EOs are obtained by steam distillation, while the reference method for *Citrus limon* oil extraction is cold pressing [23]. The extraction method plays a critical role in determining the chemical composition, quality, and bioactivity of EOs [24]. Ensuring the quality and consistency of essential oils, especially when intended for pharmaceutical or therapeutic use, requires adherence to established standards. The European Pharmacopoeia monographs provide comprehensive guidelines for the quality control of essential oils, including specifications for botanical origin, extraction method, chemical composition, and purity criteria [25]. These monographs are essential tools for verifying the authenticity and standardization of essential oils, ensuring they meet pharmaceutical-grade quality standards [26]. Compliance with these standards is particularly crucial in the development of essential oil-based formulations for medical applications, where reproducibility, safety, and efficacy are mandatory.

Essential oils rich in phenolic compounds like *Syzygium aromaticum* (clove) and *Origanum vulgare* (oregano), demonstrated potent activity against the tested bacterial strains. These oils are characterized by the presence of eugenol or carvacrol/thymol, compounds with well-documented membrane-disrupting, protein-denaturing, and anti-quorum sensing properties [9,27,28]. The chemical composition of the Caryophylli floris aetheroleum (clove oil) analyzed in this study falls within the quality specifications established by the European Pharmacopoeia, particularly with respect to the content of eugenol and β-caryophyllene [25]. In the present study, clove oil showed antibacterial activity against all tested bacterial strains with MIC/MBC values in the range of 0.4–3.125%. Similar MIC values for *S. aureus* were also reported by Xu et al. and Bai et al. [29,30]. Origani aetheroleum (oregano oil) contains more than 70% oxygenated monoterpenes, as well as peppermint EO. Two of the primary constituents are carvacrol and thymol, which collectively constitute between 40–80% of the oil, depending on geographical origin of the plant and environmental factors [31,32]. Oregano EO has gained considerable attention for its broad-spectrum antimicrobial activity, effective against bacteria, fungi, and even certain viruses. Carvacrol and thymol disrupt the cell membranes of pathogenic microbes, causing cellular contents to leak and leading to cell death [33]. The antimicrobial properties of oregano essential oil also extend to antibiotic-resistant bacteria. As it can be seen in Table 2 and Table 3, oregano oil has potent activity against MRSA and MSSA. Our results are similar with the results obtained with other *Oregano* sp. essential oils. For instance, in a study by Nostro et al. (2004), the essential oil demonstrated activity against methicillin-resistant *Staphylococcus aureus* (MRSA), a common hospital-acquired infection that is often difficult to treat with conventional antibiotics [11]. Also, the MIC and MBC values obtained for *Origanum vulgare* EO are similar with the values obtained by Man et al. on the same bacterial strains [34].

Essential oils rich in oxygenated terpenes had different antibacterial profiles, suggesting that the activity is strictly correlated with the compounds rather than the chemical class itself. This is exemplified by the diverse outcomes observed among *Salvia officinalis*, *Mentha* × *piperita*, *Rosmarinus officinalis*, and *Melaleuca alternifolia* oils, all of which are abundant in oxygenated monoterpenes. Menthol, the main monoterpenoid found in *Mentha* × *piperita*, is responsible for the characteristic cooling sensation of peppermint oil and comprises around 35–50% of its total content. Interestingly, out of the all essential oils, Menthae aetheroleum was the least potent on the tested bacterial strains, besides *Curcuma longa* EO. However, other research studies have demonstrated that peppermint oil has antibacterial activity on Gram-positive and Gram-negative bacteria at lower MIC concentrations [13,35]. These differences between our results and other published studies can be attributed to variations in chemical composition, but also to the solubilization method used in the process of sample preparation. The primary constituents of *Rosmarinus officinalis* essential oil include 1,8-cineole (also known as eucalyptol), *α*-pinene, and camphor. These compounds are mainly responsible for the oil’s bioactivity and typically comprise between 70–90% of the oil [15,36]. The main compounds found in the *Salvia officinalis* essential oil are α and β-thujone, camphor, borneol, and 1,8-cineol, which contribute to the oil’s distinct aroma and therapeutic properties [37,38]. However, the internal use of sage essential oil is not recommended due to the high content of thujone, a monoterpene ketone known to have neurotoxic effects when consumed in large quantities. Thujone can interfere with the central nervous system by acting as a GABA receptor antagonist, potentially causing symptoms such as convulsions, restlessness, and hallucinations. As a result, regulatory guidelines and health professionals advise against oral consumption of sage essential oil to prevent toxic effects. The daily dose exposure is of 6 mg/person for a maximum period of 2 weeks according to the Committee of Herbal Medicinal Products (HMPC) and the tolerable daily intake is 10 µg/kg body weight/day [39]. Nevertheless, external applications, including use in aromatherapy, skin care, and topical preparations, are generally considered safe when used appropriately and within recommended concentrations. Tea tree oil, derived from *Melaleuca alternifolia* presents potent antimicrobial, anti-inflammatory, and antifungal properties. Its main active component, terpinen-4-ol, is responsible for much of its biological activity as it increases permeability of bacterial membrane leading to leakage of intracellular components and disruption of electron transport and ion homeostasis [40]. The concentration of terpinen-4-ol in our sample is in concordance with the literature data that show a 30–48% in the composition of TTO.

Out of the studied essential oils, *Citrus limon* oil is the only one with an extremely high content in monoterpene hydrocarbons. According to the European Pharmacopoeia, the oil must contain limonene (29.5–84.7%), β-pinene (10–25.4%), γ-terpinene (~10.3%), and citral (3–5%), a profile similar with our results obtained in the GC-MS analysis [25]. Despite the well-known antimicrobial properties of these terpenes, the antibacterial activity was moderate with respect to the MIC and MBC values, and the biofilm inhibition of lemon oil was limited. This modest efficacy can be explained by the lack of polar functional groups, which reduces their ability to disrupt membrane integrity [41].

In most cases, essential oils that exhibited low MIC and MBC values, indicative of strong antibacterial activity, also showed inhibition of biofilm formation. This suggests that the antimicrobial constituents responsible for bacterial growth inhibition may concurrently interfere with biofilm development, either by preventing initial bacterial adhesion or by disrupting biofilm maturation [28,42]. However, several notable exceptions were observed. Specifically, while *Origanum vulgare* and *Rosmarinus off.* essential oils demonstrated low MIC and MBC values against methicillin-resistant *Staphylococcus aureus* (MRSA), they did not exhibit significant inhibitory effects on MRSA biofilm formation. This discrepancy highlights the complexity of biofilm physiology, as the mechanisms governing planktonic cell susceptibility can differ substantially from those involved in biofilm formation and maintenance. It is possible that while these essential oils are effective in inhibiting the growth of free-floating MRSA cells, they may lack specific compounds capable of penetrating or disrupting the biofilm matrix, or interfering with quorum sensing pathways that regulate biofilm development [28]. A similar observation was made with clove essential oil against *Pseudomonas aeruginosa*. Despite demonstrating good MIC and MBC values indicative of potent antibacterial activity, clove oil did not inhibit *P. aeruginosa* biofilm formation at lower concentrations. *P. aeruginosa* is well-known for its ability to form highly structured and protective biofilms, which can shield bacterial cells from both antimicrobial agents and host defenses. The lack of antibiofilm activity in this case suggests that clove oil’s active components may be more effective against planktonic cells but are insufficient to disrupt the complex architecture or signaling pathways essential for biofilm development in *P. aeruginosa* [43]. These findings emphasize the need to distinguish between antibacterial and antibiofilm activities when evaluating the therapeutic potential of essential oils. An essential oil with strong bactericidal properties does not necessarily guarantee efficacy against biofilm-associated infections. This distinction is particularly important in the context of oral infections, where biofilms play a central role in chronic infection and resistance to conventional therapies. Taken together, these results demonstrate the potential of specific essential oils, particularly tea tree, oregano, and lemon, as effective agents in inhibiting biofilm formation by both Gram-positive and Gram-negative bacterial pathogens. Their activity against multidrug-resistant strains, such as MRSA and *P. aeruginosa*, further indicates their potential application in preventing biofilm-associated infections, and the perspective of incorporating these EOs in mouthwashes, sprays, or dental gels. As it has been previously shown, EOs act as bactericidals through different mechanisms of action. The most notable one is the disruption of the bacterial cell membrane, due to their lipophilicity [2,12,44]. Another important mechanism is the interference with bacterial metabolic pathways, thus inhibiting bacterial cell growth [27,45]. Compared to the results obtained by Prabuseenivasan et al., our study demonstrated that the Gram-positive bacteria were more susceptible to the essential oils, rather than the Gram-negative bacteria [46].

Clustering patterns correspond in part with antimicrobial activity data, indicating that oils with similar chemical composition often exhibit comparable antibacterial or antibiofilm effects. However, the heatmap does not capture minor constituents that may act in synergy to the bioactivity.

### 3.1. Translational Aspects: Safety Thresholds and Delivery Considerations for the Topical Oral Use of Essential Oils

The results obtained in the biofilm growth assay indicated that the biofilm inhibition by essential oils was mostly seen in high concentrations that cannot be used regularly in products. Although direct cytotoxicity assays (e.g., MTT, LDH release) were not included in the present study, an in silico toxicity assessment was conducted for the major bioactive compounds using the ProTox-3.0. Most compounds, including terpinen-4-ol, menthol, and eucalyptol, were predicted to belong to toxicity classes IV–V and showed reasonable LD_50_ values, indicating low to moderate toxicity.

Additionally, published in vitro studies have demonstrated that compounds, such as eugenol and carvacrol, exhibit dose-dependent cytotoxic effects on oral epithelial and fibroblast cells, depending on the cell type and exposure time [47,48]. Notably, the MICs observed in our study for the most effective EOs (e.g., oregano, clove, tea tree) often exceeded the cytotoxic concentrations, highlighting the narrow therapeutic window of essential oils when used topically.

Translation into oral care products requires rigorous evaluation of their cytotoxicity and mucosal tolerance. The oral mucosa is more permeable and sensitive than skin, and prolonged exposure to high EO concentrations may cause epithelial damage or irritation. A comparison between the effective in vitro concentrations determined in our study and non-cytotoxic threshold reported in the literature is shown in Table 5. Although these findings suggest that therapeutically effective concentrations may exceed safety thresholds, cytotoxicity tests usually imply prolonged exposure (e.g., 24 h), whereas oral exposure is usually brief (30 s to 2 min for mouthwashes); also, in real-life conditions, saliva continuously dilutes any topical agent.

### 3.2. Limitations of the Study

A key limitation of this study is the lack of technical replication in the GC-MS analysis, which restricts the statistical strength of the clustering results. Nevertheless, the data offer valuable insight into compositional trends among commonly used essential oils and suggest that future studies should incorporate analytical and biological in vivo replicates to validate and expand upon these findings.

The current study focused on evaluating the antibacterial and antibiofilm activity of essential oils against five facultative bacterial strains not classically associated with periodontal disease: *Staphylococcus aureus*, *Enterococcus faecalis*, *Pseudomonas aeruginosa*, *Escherichia coli*, and *Klebsiella pneumoniae*. We acknowledge that these strains are not considered periodontal pathogens. However, these species have been frequently detected in the oral cavity, mostly in individuals with poor oral hygiene, mucosal inflammation, etc. Their ability to form biofilms and develop antimicrobial resistance supports the inclusion of these bacterial strains in models of oral dysbiosis and localized infections. Future studies should include strict anaerobic cultures, as well as consider multi-species biofilm, and assessment of cytotoxicity in vitro.

## 4. Materials and Methods

### 4.1. Samples

The essential oils used in this study were commercially existing essential oils obtained by steam distillation of clove, peppermint, oregano, rosemary, sage, tea tree, and turmeric. Lemon oil was obtained by mechanical process. The extraction method for each essential oil was specified on the supplier’s certificate of analysis. From each oil, a sample was deposited at the Department of Pharmacognosy and Phytotherapy as follows: RS-C-CEO-24, RS-C-PEO-24, RS-C-OEO-24, RS-C-REO-24, RS-C-TTEO-24, RS-C-TEO-24.

### 4.2. GC-MS Analysis

The gas chromatography–mass spectrometry (GC-MS) analysis was performed on a 7890B GC-5977A MSD system (Agilent Technologies, Santa Clara, CA, USA) equipped with an HP-5MS UI, 30 m × 0.25 mm, 0.25 µm (Agilent Technologies) column. Helium was used as the carrier gas with a flow rate of 1 mL/min. The oven program was set to 60 °C (1 min), 60–250 °C ramp (10 °C/min), hold 250 °C (5 min). Spitless injection, 1 µL sample volume injected into the column. MS detection parameters: scan mode, in the range of 40–500 *m*/*z*. MS spectra analysis was performed using NIST14 and MPW5e (version 14) (Wiley libraries, Hoboken, NJ, USA). Sample preparation: essential oil samples were diluted 400-fold with *n*-hexane (GC-MS purity). For the identification of volatile compounds, Kováts retention indices (RI) were calculated based on the retention times of a homologous series of n-alkanes (C_10_ to C_26_) under the same chromatographic conditions as those used for sample analysis.

### 4.3. Bacterial Strains

The antibacterial activity was evaluated on 5 different strains (*Staphylococcus aureus* MSSA ATCC 29213, methicillin-resistant *Staphylococcus aureus* (MRSA) ATCC 43300, *Pseudomonas aeruginosa* ATCC 27853, *Escherichia coli* ATCC 25922, and *Klebsiella pneumoniae* ATCC 700603) from the collection of the Microbiology Department of the George Emil Palade University of Medicine, Pharmacy, Sciences, and Technology of Târgu-Mureș.

### 4.4. Antimicrobial Activity Evaluation

The EOs were diluted using a previously described method [6]. Briefly, each essential oil was diluted in water after being solubilized with Tween 20, and sonicated for 10 min. The MIC (Minimum Inhibitory Concentration) of each EO was assessed by the broth micro-dilution method, according to the EUCAST recommendations [56]. Briefly, 0.5 McFarland inoculi were created in a sterile saline solution. From each inoculum, 10 μL were transferred in Muller Hinton broth 2X (stock solution with a final volume of 10 mL). In sterile microtiter plates, the EOSs were serially diluted in sterile water. On each well, stock solution was added (final volume/each well of 200 μL), and the concentration range of each oil was 0.02–6.25%. The plates were incubated for 24 h at 37 °C. Positive control wells (culture media with bacteria) and negative control wells (only culture media) were used. The experiments were conducted in triplicate. After incubation, the bacterial growth was visually assessed, and MIC was defined as the lowest concentration of essential oil at which no visible bacterial growth (i.e., clear well, no turbidity) was observed.

The MBC (minimum bactericidal concentration) was determined by culturing 1 μL from each well with no bacterial growth on Muller Hinton Agar. The plates were incubated at 37 °C for 18 h and, after incubation, visually inspected for bacterial growth.

### 4.5. Biofilm Growth Assay

From fresh cultures of the above-mentioned reference strains, 0.5 McFarland inoculi were created in a sterile saline solution, corresponding to a concentration of bacteria of aprox. 1.5 × 10^8^ CFU/mL. The density of each inoculi was measured using DEN-1 McFarland Densitometer (Biosan, Saratoga Springs, NY, USA). From each inoculum, 10 μL were mixed with nutrient broth. The EOs were serially diluted in sterile water, in sterile microtiter plates. Biofilm inhibition was evaluated using EO concentrations ranging from 6.25 to 50%. On each well, 100 μL of the bacteria with nutrient broth were added (wells’ final volume of 200 μL). The plates were incubated (37 °C, 24 h) to allow the bacteria to form biofilms. After incubation, the biofilms were gently washed twice to remove planktonic and loosely attached cells and then stained (using the crystal violet staining technique). Briefly, 200 µL of 0.1% (*w*/*v*) crystal violet solution was added to each well and the plates were incubated for 15 min at room temperature. Excess stain was removed by gently washing the wells three times with sterile distilled water, and the plates were allowed to air-dry completely. Subsequently, 200 µL of 30% (*v*/*v*) acetic acid was added to each well to solubilize the bound crystal violet, and the plates were incubated and shacked for an additional 15 min at 25 °C using a Thermo-Shaker (Biosan, Riga, Latvia) [57]. Positive and negative controls were used. The OD (Optical Density) of each sample was spectrophotometrically assed on a multiplate reader (Epoch, Biotek, Shoreline, WA, USA) at a wavelength of 620 nm. The EO concentrations were selected based on preliminary assays and also based on similar studies evaluating antibacterial activity of essential oils [58,59].

Biofilm inhibition was calculated using the following formula:

$$Biofilm\;inhibition\;(\%)=\frac{{OD}_{control}-{OD}_{test}}{{OD}_{control}}\times 100$$

As an additional indicator, the delta index (ΔI) was calculated as the ratio between treated and untreated biofilm. A Δ Index ≥ 1.25 stands for biofilm formation stimulation.

### 4.6. In Silico Toxicity Prediction

In order to assess the safety profile of major constituents identified in the essential oils, an in silico toxicity analysis was performed using ProTox-3.0. The following parameters were predicted: LD_50_, toxicity class (ranging from Class I–extremely toxic to Class V–non-toxic).

### 4.7. Statistical Analysis

The chemical composition data obtained from the gas chromatography-mass spectrometry (GC-MS) analysis of the eight essential oils were expressed as relative peak areas (%). To identify the similarities and differences among the essential oils based on their chemical profiles, multivariate statistical analyses were performed.

A hierarchical clustering analysis (HCA) was performed using the Ward’s linkage method with Euclidean distance as the similarity measure. The results were visualized in a clustered heatmap, allowing for the simultaneous display of the relative abundance of each compound and the clustering relationships between essential oils and their constituent compounds. All statistical analyses and visualizations were performed using Python 3.11 (Python Software Foundation, Beaverton, OR, USA).

## 5. Conclusions

This study demonstrates that several essential oils exhibit notable antibacterial and antibiofilm activity against non-periodontal pathogens associated with oral dysbiosis. *Origanum vulgare*, *Melaleuca alternifolia*, *Syzygium aromaticum*, and *Rosmarinus officinalis* oils displayed the best inhibitory activity, in both assays. These effects were linked in the majority of cases to the presence of oxygenated monoterpenes and phenolic compounds in the essential oils. Our findings support the inclusion of specific EOs as bioactive ingredients in oral hygiene products aimed at preventing or managing dysbiosis. However, this study focused on single-species biofilms and in vitro models. While this provides mechanistic insight, our study does not fully present the complexity of polymicrobial oral biofilms or host–pathogen interactions. Future research should focus on evaluating these EOs in multispecies biofilm systems and assessing their safety and efficacy in vivo. Also, standardization of EO composition and formulation strategies are very important steps for translation into clinical use.

## Figures and Tables

**Table 1:** Chemical composition of the studied EOs.

Rt (min)	Compound	Molecular Mass	RI	RIL	Clove	Lemon	Oregano	Peppermint	Rosemary	Sage	Tea Tree	Turmeric
		Peak Area (%)										
4.107	*α*-Pinene	C_10_H_16_	928	928	-	11.6	-	0.6	20.14	4.13	1.66	-
4.31	Cyclofenchene	C_10_H_16_	943	946	-	-	-	-	9.24	-	-	-
4.313	Camphene	C_10_H_16_	946	948	-	-	-	-	-	6.46	-	-
4.694	Sabinene	C_10_H_16_	970	974	-	-	-	-	-	-	0.91	-
4.739	*β*-Phellandrene	C_10_H_20_	974	1005	-	2.21	-	0.13	-	-	0.16	-
4.887	*β*-Pinene	C_10_H_16_	984	974	-	4.68	1.91	1.33	4.22	3.61	-	-
5.114	*α*-Phellandrene	C_10_H_20_	1001	1004	-	-	-	-	0.16	-	1.15	0.42
5.156	*3*-Carene	C_10_H_16_	1004	1008	-	0.14	-	0.18	-	-	-	-
5.252	2-Carene	C_10_H_16_	1011	1003	-	-	-	-	-	-	9.8	-
5.289	*α*-Terpinene	C_10_H_16_	1014	1017	-	1.04	-	-	-	-	-	-
5.373	*o*-Cymene	C_10_H_14_	1020	1026	-	-	7.29	tr	1.75	0.18	-	0.69
5.488	D-Limonene	C_10_H_16_	1024	1029	-	52.91	-	2.11	4.41	0.64	23.9	-
5.509	Eucalyptol	C_10_H_18_O	1028	1031	-	-	-	11.45	23.11	10.27	-	0.8
5.87	*γ*-Terpinene	C_10_H_16_	1055	1059	-	4.62	10.2	-	2.32	1.09	16.41	tr
6.314	Terpinolene	C_10_H_18_	1088	1087	-	5.95	-	-	-	-	-	-
6.324	Carvomenthene	C_10_H_18_	1089	1023	-	-	-	-	-	0.7	-	-
6.468	Linalool	C_10_H_18_O	1101	1099	-	0.98	-	0.57	2.11	0.56	0.15	-
6.653	Thujone	C_10_H_16_O	1112	1114	-	-	-	-	-	19.83	-	-
6.72	Fenchol	C_10_H_18_O	1117	1115	-	0.11	-	-	-	-	tr	-
7.236	Camphor	C_10_H_16_O	1154	1143	-	-	-	-	2.32	-	-	-
7.253	Citronellal	C_10_H_18_O	1155	1153	-	0.32	-	-	-	-	-	-
7.262	(+)-2-Bornanone	C_10_H_16_O	1155	1149	-	-	-	-	-	11.73	-	-
7.362	Menthone	C_10_H_18_O	1162	1150	-	-	tr	28.98	-	-	-	-
7.426	Isoborneol	C_10_H_18_O	1167	1158	-	-	-	-	14.49	-	0.11	-
7.429	Isoneral	C_10_H_16_O	1167	1165	-	0.68	-	-	-	-	-	-
7.528	Endo-Borneol	C_10_H_18_O	1174	1166	-	-	-	-	0.29	3.39	0.53	-
7.681	Isogeranial	C_10_H_16_O	1185	1185	-	1.23	-	-	-	-	-	-
7.728	Levomenthol	C_10_H_20_O	1188	1177	-	-	-	38.8	-	-	-	-
7.76	Terpinen-4-ol	C_10_H_18_O	1191	1177	-	-	-	-	0.59	0.51	31.56	tr
7.87	*α*-Terpineol	C_10_H_18_O	1198	1189	-	0.44	-	-	5.11	0.15	2.7	-
8.257	*cis*-Carveol	C_10_H_16_O	1226	1227		0.15						
8.583	Pulegone	C_10_H_16_O	1252	1234	-	-	-	0.56	-	-	-	-
8.643	D-carvone	C_10_H_14_O	1256	1242	-	0.11	tr	1.24	-	-	-	-
8.701	Geraniol	C_10_H_18_O	1261	1252	-	0.22	-	-	-	-	-	-
8.703	Linalyl acetate	C_12_H_20_O_2_	1261	1257	-	-	-	0.65	-	-	-	-
8.754	Chavicol	C_10_H_14_O_3_	1265	1252	0.25	-	-	-	-	-	-	-
8.967	Neral	C_10_H_16_O	1280	1238	-	4.33	-	-	-	-	-	-
9.197	Bornyl acetate	C_12_H_20_O_2_	1298	1283	-	-	-	-	-	1.77	-	-
9.227	Isobornyl acetate	C_12_H_20_O_2_	1300	1284	-	-	-	-	2.84	-	-	-
9.254	Thymol	C_10_H_14_O	1302	1290	-	-	11.33	-	-	-	-	0.14
9.29	Menthyl acetate	C_12_H_22_O_2_	1305	1295	-	-	-	6.5	-	-	-	-
9.528	Carvacrol	C_10_H_14_O	1322	1300	-	-	65.95	-	-	-	-	-
10.042	*α*-Cubebene	C_15_H_24_	1359	1351	-	-	-	0.11	-	tr	-	-
10.344	Eugenol	C_10_H_12_O	1381	1357	79.75	-	-	-	-	-	-	-
10.418	*α*-Copaene	C_15_H_24_	1387	1376	-	-	-	0.26	tr	-	tr	-
10.43	Geranyl acetate	C_12_H_20_O_2_	1387	1379	-	0.43	-	-	-	-	-	-
10.599	Germacrene D	C_15_H_24_	1400	1480	-	tr	-	-	-	-	-	-
10.745	Sesquithujene	C_15_H_24_	1411	1410	-	-	-	-	-	-	-	tr
10.772	Methyleugenol	C_11_H_14_O_2_	1414	1401	0.19	-	-	-	-	-	-	-
11.03	*β*-Caryophyllene	C_15_H_24_	1436	1420	11.42	0.24	-	3.22	1.41	5.26	tr	1.22
11.128	*β*-Copaene	C_15_H_24_	1442	1433	-	tr	-	-	-	-	-	-
11.269	Aromadendrene	C_15_H_24_	1453	1440	-	-	-	tr	-	tr	tr	-
11.406	*trans*-Isoeugenol	C_10_H_12_O_2_	1464	1454	0.12	-	-	-	-	-	-	-
11.475	Humullene	C_15_H_24_	1469	1454	2.37	-	-	0.14	-	5.3	-	0.31
11.709	*γ*-Curcumene	C_15_H_22_	1488	1480	-	-	-	-	-	-	-	1.99
11.768	*α*-Curcumene	C_15_H_22_	1493	1482	-	-	-	-	-	-	-	6.4
11.879	*β*-Humullene	C_15_H_24_	1502	1442	-	-	-	-	-	tr	-	-
11.909	*α*-Zingiberene	C_15_H_24_	1504	1495	-	-	-	-	-	-	-	5.01
12.013	*α*-Farnesene	C_15_H_24_	1513	1504	0.18	-	-	-	-	-	-	-
12.061	*β*-Bisabolene	C_15_H_24_	1517	1508	-	-	-	-	-	-	-	2.03
12.1	*β*-Curcumene	C_15_H_22_	1520	1512	-	-	-	-	-	-	-	0.41
12.268	*β*-Sesquiphellandrene	C_15_H_24_	1534	1523	-	-	-	-	-	-	-	3.44
12.284	Cadinene	C_15_H_24_	1535	1533	tr	-	-	-	-	-	-	-
12.297	*trans*-Calamenene	C_15_H_22_	1536	1528	-	-	-	-	-	tr	-	-
12.332	Dihydro-eugenol acetate	C_12_H_16_O	1539	1536	0.24	-	-	-	-	-	-	-
12.462	*α*-Bisabolene	C_15_H_24_	1550	1540	-	tr	-	-	-	-	-	-
12.930	Caryophyllenyl alcohol	C_15_H_26_O	1603	1580	0.28	-	-	-	-	-	-	-
12.973	*cis*-Nuciferol	C_15_H_22_O	1593	1744	-	-	-	-	-	-	-	0.6
13.09	Caryophyllene oxide	C_15_H_24_O	1603	1580	1.21	-	0.54	0.54	tr	0.21	-	-
13.182	Globulol	C_15_H_26_O	1611	1581	-	-	-	-	-	0.24	-	-
13.275	*α*-Santalol	C_15_H_24_O	1619	1683	0.14	-	-	-	-	-	-	-
13.92	*α*-Tumerone	C_15_H_22_O	1676	1668	-	-	-	-	-	-	-	10.41
14.051	Ar-Turmerone	C_15_H_20_O	1687	1668	-	-	-	-	-	-	-	26.52
14.284	*γ*-Atlantone	C_15_H_22_O	1708	1679	-	-	-	-	-	-	-	6.2
14.399	*β*-Tumerone	C_15_H_22_O	1718	1699	-	-	-	-	-	-	-	4.94
14.796	(6*R*,7*R*)-Bisabolone	C_15_H_24_O	1753	1742	-	-	-	-	-	-	-	0.79
15.165	(*E*)-Atlantone	C_15_H_22_O	1785	1775	-	-	-	-	-	-	-	0.93
Total					96.15	92.39	97.22	97.37	94.51	76.03	89.04	73.25
Monoterpene hydrocarbons					-	83.15	19.4	4.35	42.24	16.81	53.99	1.11
Oxigenated monoterpenes					-	9	77.28	88.75	50.86	48.21	35.05	0.94
Sesquiterpenes hydrocarbons					13.97	0.24	-	3.73	1.41	10.56	-	21.08
Oxigenated sesquiterpenes					1.63	-	0.54	0.54	-	0.45	-	50.39
Phenylpropanoid derivatives					80.55	-	-	-	-	-	-	-

tr—trace amounts, <0.1; the retention index (RI) was calculated using a homologous series of n-alkanes C_8_–C_18_. RIL—literature retention index [16,17].

**Table 2:** Antibacterial activity (MIC and MBC in % essential oil).

Essential Oil	MIC/MBC				
	MSSA (ATCC 29213)	MRSA (ATCC 43300)	*P. aeruginosa* (ATCC 27853)	*E. coli* (ATCC 25922)	*K. pneumoniae* (ATCC 700603)
Sage	0.4%/0.4%	3.125%/3.125%	3.125%/3.125%	3.125%/3.125%	-
Turmeric	3.125%/-	3.125%/-	-	-	-
Oregano	0.4%/0.4%	0.05%/3.125%	3.125%/3.125%	0.05%/0.05%	3.125%/3.125%
Clove	3.125%/3.125%	0.4%/0.4%	0.4%/0.4%	0.4%/0.4%	3.125%/3.125%
Lemon	3.125%/-	3.125%/3.125%	-	3.125%/3.125%	3.125%
Peppermint	3.125%/-	-	-	3.125%	-
Rosemary	0.05%/0.4%	0.05%/3.125%	3.125%/3.125%	0.4%/3.125%	3.125%/3.125%
Tea tree	0.05%/0.05%	0.05%/0.05%	0.4%/0.4%	0.4%/0.4%	0.4%/3.125%

Values are mean inhibition concentration of three replicates; no activity was observed within the tested concentration range (≤6.25%).

**Table 3:** Δ Index and biofilm inhibition.

Essential Oil	Concentration (%)	MSSA	MRSA	*P. aeruginosa*	*E. coli*	*K. pneumoniae*
		BI	BI	BI	BI	BI
Sage	50	++	+++	+++	+/−	++
	25	++	++	+++	+/−	+/−
	12.5	+/−	+/−	++	↑	+/−
	6.25	↑	+/−	↑	↑	+/−
Turmeric	50	++	++	++	↑	↑
	25	++	++	++	↑	↑
	12.5	++	++	++	↑	↑
	6.25	+/−	++	++	↑	↑
Oregano	50	+++	++	+++	+/−	++
	25	++	+/−	+++	+/−	+/−
	12.5	+/−	+/−	+++	+/−	+/−
	6.25	+/−	+/−	++	+/−	+/−
Clove	50	+++	+++	+++	++	++
	25	+++	+++	++	++	++
	12.5	+++	+++	+/−	++	++
	6.25	++	++	+/−	++	+++
Lemon	50	+++	+++	+++	++	++
	25	++	+++	+++	++	++
	12.5	++	+/−	+++	↑	+/−
	6.25	+/−	+/−	+++	↑	+/−
Peppermint	50	+++	+++	+++	++	+/−
	25	+++	+++	+++	+/−	+/−
	12.5	++	++	+++	+/−	+/−
	6.25	+/−	+/−	+++	↑	↑
Rosemary	50	+++	+++	+++	++	++
	25	+++	+++	+++	++	+/−
	12.5	++	++	++	+/−	+/−
	6.25	+/−	+/−	++	+/−	+/−
Tea tree	50	++	++	++	++	+/−
	25	+/−	+/−	+/−	+/−	↑
	12.5	+/−	↑	↑	+/−	↑
	6.25	↑	↑	↑	↑	↑

Symbols represent biofilm-modulating activity: +++ = strong inhibition (≥75%); ++ = moderate inhibition (40–74%); +/− = weak/no effect (<40%); ↑ = biofilm stimulation (ΔI > 1.25 or negative inhibition %).

**Table 4:** Oral toxicity prediction for the main compounds found in the selected essential oils.

Essential Oil	MIC % (*v*/*v*)	Main Bioactive Compound	Predicted LD_50_ (mg/kg)	Toxicity Class	Toxicological Endpoints Target	Comments
Sage	0.4%	Thujone	500	IV	Neurotoxicity, BBB	Use restricted in cosmetics and food products due to neurotoxicity; regulated under EU Regulation 1334/2008; TDI = 10 µg/kg body weight/day [18]
Turmeric	3.125%	ar-Turmerone	2000	IV	BBB	Limited toxicity data
Oregano	0.4%	Carvacrol	810	IV	BBB, Mitochondrial membrane potential, CYP2C9	Potent antimicrobial activity; may irritate skin or mucosa at high concentrations [19]
Clove	3.125%	Eugenol	650	IV	Neurotoxicity, BBB	Commonly used in dentistry as analgesic and antiseptic; recognized for safe usage at low concentrations [20]
Lemon	3.125%	Limonene	4400	V	Cardiotoxicity, BBB, CYP2C9	Widely used in cosmetics and fragrances; potential skin sensitizer; generally recognized as safe by FDA
Peppermint	3.125%	Menthol	940	IV	BBB	Approved for oral and topical used; cooling agent in OTC products; can produce mucosal irritation in high doses
Rosemary	0.05%	Eucalyptol	2480	V	BBB	Common in mouthwashes and cough syrups; approved for use in cosmetics and oral hygiene products
Tea tree	0.05%	Terpinen-4-ol	1016	IV	BBB	Well-tolerated in topical formulations at <1%; cytotoxicity at higher concentrations [21]

TDI—tolerable daily intake; BBB—blood–brain barrier.

**Table 5:** Overview of safe concentrations and formulation recommendations for the selected essential oils.

Essential Oil/Major Compound	MIC % (*v*/*v*)	Non-Cytotoxic Level (%)/Cell Line ^#^	Non-Irritant Dermal Concentrations (%) *	Suggested Delivery Formulation	Observations	References
Sage (thujone)	0.4%	0.03%/TR146	recommended concentration: ≤0.4%	short contact rinse; synergistic formulation mouthwash	thujone is convulsant and neurotoxic; avoid internal use	[49,50,51]
Turmeric (ar-Turmerone)	3.125%	n.d.	non-irritant concentrations: 4%	controlled delivery formulations; flavoring; recommended concentrations: ≤2%	limited data on oral safety	[51]
Oregano (carvacrol)	0.4%	0.005–0.008%/L969, HGF	non-irritant concentrations: 2%	diluted mouthwash; short exposure; recommended concentrations: <1%	highly irritant if used undiluted	[51,52]
Clove (eugenol)	3.125%	Eugenol cytotoxicity: 0.01% after 24 h exposure	non-irritant concentrations: <3%	root canal use; diluted mouthwash; lozenges; synergistic with clorhexidine; recommended concentration: <1%	eugenol is frequently used in dental products in concentrations of 0.05–0.1%	[51,53]
Lemon (limonene)	3.125%	n.d.	non-irritant concentrations: 2–5%;	diluted mouthwash; flavoring; recommended concentrations: ≤2%	oxidation products of limonene can cause skin sensitization	[51]
Peppermint (menthol)	3.125%	0.01%/HaCaT	non-irritant concentrations: <10%; low overall toxicity	mouthwash, hydrogels, lozenges, spray	has GRAS status; frequently used in dental products; pulegone content should be <1%	[51,54]
Rosemary (eucalyptol)	0.05%	n.d.	non-irritant concentrations: <10%	mouthwash, hydrogels, low-dose lozenges, spray	generally well tolerated	[51]
Tea tree (terpinen-4-ol)	0.05%	≤0.03%/HaCaT, HGF	10–20%	mouthwash, hydrogels, not lozenges; recommended concentration: 2.5%	tea tree oil is prone to oxidation	[51,55]

^#^ data from in vitro studies; * data from in vivo studies; n.d.—no data found in the searched databases; HGF—human gingival fibroblast; GRAS—generally recognized as safe.

## Data Availability

The original contributions presented in this study are included in the article. Further inquiries can be directed to the corresponding author.
